# Association of impaired fasting glucose, diabetes and their management with the presentation and outcome of peripheral artery disease: a cohort study

**DOI:** 10.1186/s12933-014-0147-2

**Published:** 2014-11-01

**Authors:** Jonathan Golledge, Frank Quigley, Ramesh Velu, Phillip J Walker, Joseph V Moxon

**Affiliations:** Queensland Research Centre for Peripheral Vascular Disease, School of Medicine and Dentistry, James Cook University, Townsville, Australia 4811; Department of Vascular and Endovascular Surgery, The Townsville Hospital, Townsville, Australia; Discipline of Surgery and Centre for Clinical Research, University of Queensland School of Medicine, Brisbane, Australia; Department of Vascular Surgery, The Royal Brisbane and Women’s Hospital, Brisbane, Australia

**Keywords:** Peripheral artery disease, Diabetes, Mortality, Surgery, Patient management

## Abstract

**Background:**

Pre-diabetes and untreated diabetes are common in patients with peripheral artery disease however their impact on outcome has not been evaluated. We examined the association of impaired fasting glucose, diabetes and their treatment with the presentation, mortality and requirement for intervention in peripheral artery disease patients.

**Methods:**

We prospectively recruited 1637 patients with peripheral artery disease, measured fasting glucose, recorded medications for diabetes and categorised them by diabetes status. Patients were followed for a median of 1.7 years.

**Results:**

At entry 22.7% patients were receiving treatment for type 2 diabetes by oral hypoglycaemics alone (18.1%) or insulin (4.6%). 9.2% patients had non-medicated diabetes. 28.1% of patients had impaired fasting glucose (5.6-6.9 mM). Patients with non-medicated diabetes had increased mortality and requirement for peripheral artery intervention (hazards ratio 1.62 and 1.31 respectively). Patients with diabetes prescribed insulin had increased mortality (hazard ratio 1.97). Patients with impaired fasting glucose or diabetes prescribed oral hypoglycaemics only had similar outcomes to patients with no diabetes.

**Conclusions:**

Non-medicated diabetes is common in peripheral artery disease patients and associated with poor outcomes. Impaired fasting glucose is also common but does not increase intermediate term complications. Peripheral artery disease patients with diabetes requiring insulin are at high risk of intermediate term mortality.

**Electronic supplementary material:**

The online version of this article (doi:10.1186/s12933-014-0147-2) contains supplementary material, which is available to authorized users.

## Background

Peripheral artery disease (PAD) represents a collection of occlusive and aneurysmal diseases affecting the peripheral arteries with an associated high risk of major cardiovascular events and mortality [1]. There are recognised deficiencies in current methods of predicting and limiting complications in PAD patients [2,3]. Globally, an estimated 382 million people have diabetes and this number will rise to 592 million in 2035 [4]. Diabetes is a recognised risk factor for occlusive PAD development but is negatively associated with peripheral aneurysm development [5-8]. Diabetes has been independently associated with mortality in PAD patients in some but not all studies [9-13]. Recent data suggest that ~10% of patients presenting with PAD have untreated diabetes and ~17% have impaired fasting glucose [14]. The effect of impaired fasting glucose and untreated diabetes on PAD outcome is unknown. This study aimed to examine the association of impaired fasting glucose, diabetes and their treatments with the presentation, mortality and requirement for intervention of PAD patients.

## Methods

An expanded methods section is provided in the Additional file 1.

### Study design and sample size

This study was part of on-going prospective cohort investigation of peripheral vascular disease patients aimed at assessing risk predictors of peripheral vascular disease presence and outcome [15,16]. Monte-Carlo simulations suggest that a multivariate regression model is adequately powered when using 10 outcome events per degree of freedom of the predictor variable [17]. We estimated that mortality at one year would be ~10% and planned to adjust for up to 15 variables in our regression model. This suggested that ~1600 patients would provide sufficient power to examine the association of diabetes with mortality.

### Patients

Patients were recruited from in and out-patient vascular services at The Townsville Hospital, The Mater Hospital Townsville and The Royal Brisbane and Women’s Hospital, Australia. Patients with all types of peripheral vascular disease were considered for inclusion as previously described [15,18]. Inclusion criteria for the current study included a diagnosis of PAD, assessment of fasting blood glucose and at least one follow-up assessment as an in or out-patient. This study was conducted in accordance with the Declaration of Helsinki. Ethical approval for the study was granted by the local Institutional Ethics Committees at The Townsville Hospital, The Mater Hospital Townsville, The Royal Brisbane and Women’s Hospital and James Cook University (61/05, MHS2006-01, H2196, 2007/004, 12/QTHS/202, MHS20140114-01, H5206, 13/ QTHS/125). Participants provided written informed consent for inclusion.

### Definitions and diagnosis of PAD

The current study included patients with occlusive or aneurysmal disease of their peripheral arteries. Presenting complaints included asymptomatic carotid stenosis, mild lower limb or upper limb peripheral athero-thrombosis, aneurysm of the aorta or peripheral arteries, symptomatic carotid artery stenosis and critical lower limb ischaemia, as previously described [15,16,18].

### Assessment of fasting blood glucose and diabetes

Patients were asked if they were receiving medications for the treatment of diabetes, specifically oral hypoglycaemics or insulin. Patients provided blood samples after an overnight fast for automated assessment of serum glucose as part of their clinical care. Utilising clinical information and blood glucose measurements, patients were grouped as follows:

- No diabetes: Receiving no medications for diabetes and fasting blood glucose <5.6 mM;
- Impaired fasting glucose: Receiving no medications for diabetes and fasting blood glucose 5.6-6.9 mM;
- Non-medicated diabetes: Receiving no medications for diabetes and fasting blood glucose ≥7.0 mM;
- Diabetes prescribed oral hypoglycaemics only: Currently prescribed one or more oral hypoglycaemic agents but not insulin for previously diagnosed diabetes;
- Diabetes prescribed insulin: Currently prescribed insulin for previously diagnosed diabetes.

### Definitions of other risk factors

Hypertension was defined by a history of high blood pressure or receiving treatment to reduce blood pressure [15,16,18]. Smoking status was classified as ever and never smokers [15,16,18]. Coronary heart disease (CHD) was defined by a history of myocardial infarction, angina or treatment for coronary artery disease [15,16,18]. Estimated glomerular filtration rate (eGFR) was calculated using the Chronic Kidney Disease-Epidemiology Collaboration group (CKD-EPI) formula since we have previously found this to be most accurately associated with complications in PVD patients [16].

### Medications

In addition to anti-diabetic drugs, each patient’s medications were recorded including whether the participants were prescribed a statin, aspirin, another anti-platelet agent, a beta-blocker, a calcium channel blocker (CCB), an angiotensin converting enzyme (ACE) inhibitor, an angiotensin receptor blocker (ARB) and frusemide [15,16,18].

### Follow-up

Patients were followed up through attendance at out-patient clinics and /or as an in-patient as part of their normal medical care as previously described [15,16,18].

### Recording of outcome data

The primary outcome was mortality. The secondary outcome was requirement for a peripheral artery intervention. Outcome data was recorded during clinical reviews on prospectively defined report forms. Charts and hospital electronic records of all patients were reviewed by a vascular specialist or clinical researcher. For peripheral artery intervention assessments, patients were censored at the time of the first intervention or at the date of last in/out patient review or death if no intervention was required.

### Statistical analyses

Nominal data are presented as numbers and percentages. The association of diabetes categories with the clinical presentation and risk factors of the patients was assessed using Kruskal Wallis and chi-squared tests. The associations of diabetes categories with death and requirement for peripheral artery intervention were assessed using Kaplan Meier estimates, log rank test and Cox proportional hazard analyses. Cox proportional hazard analyses were adjusted for varying combinations of risk factors (age, sex, hypertension, ever smoking, CHD, presenting complaint, statin prescription, aspirin prescription, other anti-platelet prescription, beta blocker prescription, CCB prescription, ACE inhibitor prescription, ARB prescription, frusemide prescription and eGFR) across four different models. These covariates were included as they are recognised determinants of outcome for patients with cardiovascular disease.

For Cox proportional hazards analyses, diabetes categories were defined as indicators in the following order: No diabetes; impaired fasting glucose; non-medicated diabetes; diabetes prescribed oral hypoglycaemics only; and diabetes prescribed insulin. Presenting peripheral artery disease complaint was defined as indicators in the following order: asymptomatic carotid stenosis; mild lower limb or upper limb peripheral athero-thrombosis; aneurysm of the aorta or peripheral arteries; symptomatic carotid artery stenosis; and critical lower limb ischemia. Age and eGFR were included in regression models as continuous numbers. Binary variables were defined as present or absent.

## Results and discussion

### Risk factors of the cohort

1637 PAD patients were included in the current study. The presenting complaints of the patients included aortic or peripheral aneurysms (n = 707; 43.2%), mild limb peripheral artery disease (n = 381; 23.3%), symptomatic carotid artery stenosis (n = 226; 13.8%), critical limb ischaemia (n = 184; 11.2%) and asymptomatic carotid artery stenosis (n = 139; 8.5%). The median age of the patients was 71 years and approximately three quarters were male (Table 1). Risk factors for cardiovascular disease included a history of hypertension and smoking in 77% and 82%, respectively. Forty eight percent of patients had a past history of diagnosis or treatment of CHD.

**Table 1:** **Association of impaired fasting glucose, diabetes and their management with other cardiovascular risk factors, medication prescription and presentation in 1637 patients with peripheral artery disease**

	**Diabetes category**						
	**1**	**2**	**3**	**4**	**5**	**Total**	**P value**
Number	655 (40.0%)	460 (28.1%)	150 (9.2%)	296 (18.1%)	76 (4.6%)	1637	
Age (years)	70.83	71.27	71.12	70.35	65.47	70.68	0.001
	(63.28-76.98)	(64.64-76.69)	(66.19-76.91)	(64.22-75.01)	(59.74-72.07)	(64.13-76.43)	
Male	477	352	114	226	55	1224	0.591
	(72.8%)	(76.5%)	(76.0%)	(76.4%)	(72.4%)	(74.8%)	
Hypertension	470	361	105	258	65	1259	<0.001
	(71.8%)	(78.5%)	(70.0%)	(87.2%)	(85.5%)	(76.9%)	
Ever smoker	527	399	123	238	62	1349	0.074
	(80.5%)	(86.7%)	(82.0%)	(80.4%)	(81.6%)	(82.4%)	
CHD	282	216	79	155	47	779	0.003
	(43.1%)	(47.0%)	(52.7%)	(52.4%)	(61.8%)	(47.6%)	
eGFR (mL/min/1.73 m^2^)	76.39	73.37	74.22	74.93	69.17	74.93	0.040
	(59.40-90.43)*	(56.27-87.71)^†^	(51.99-87.76)^‡^	(56.88-88.96)^§^	(44.47-91.02)^||^	(56.97-89.35)	
**Presenting complaint**							
Asymptomatic carotid stenosis	56	35	11	30	7	139	0.772
	(8.5%)	(7.6%)	(7.3%)	(10.1%)	(9.2%)	(8.5%)	
Mild limb peripheral artery disease	151	91	25	89	25	381	0.001
	(23.1%)	(19.8%)	(16.7%)	(30.1%)	(32.9%)	(23.3%)	
Aortic and peripheral artery aneurysm	302	225	84	88	8	707	<0.001
	(46.1%)	(48.9%)	(56.0%)	(29.7%)	(10.5%)	(43.2%)	
Symptomatic carotid artery stenosis	90	70	15	43	8	226	0.491
	(13.7%)	(15.2%)	(10.0%)	(14.5%)	(10.5%)	(13.8%)	
Critical limb ischaemia	56	39	15	46	28	184	<0.001
	(8.5%)	(8.5%)	(10.0%)	(15.5%)	(36.8%)	(11.2%)	
**Prescribed medication**							
Statin	392	325	92	232	61	1102	<0.001
	(59.8%)	(70.7%)	(61.3%)	(78.4%)	(80.3%)	(67.3%)	
Aspirin	415	311	98	226	48	1098	0.003
	(63.4%)	(67.6%)	(65.3%)	(76.4%)	(63.2%)	(67.1%)	
Other anti-platelets	130	90	37	72	21	350	0.194
	(19.8%)	(19.6%)	(24.7%)	(24.3%)	(27.6%)	(21.4%)	
Beta-blockers	199	186	53	108	28	574	0.014
	(30.4%)	(40.4%)	(35.3%)	(36.5%)	(36.8%)	(35.1%)	
Calcium channel blocker	155	142	50	99	27	473	0.004
	(23.7%)	(30.9%)	(33.3%)	(33.4%)	(35.5%)	(28.9%)	
ACE inhibitor	227	182	58	153	52	672	<0.001
	(34.7%)	(39.6%)	(38.7%)	(51.7%)	(68.4%)	(41.1%)	
ARB	142	94	34	94	17	381	0.005
	(21.7%)	(20.4%)	(22.7%)	(31.8%)	(22.4%)	(23.3%)	
Metformin	0	0	0	245	40	285	<0.001
				(82.8%)	(52.6%)	(17.4%)	
Other oral hypoglycaemic	0	0	0	170	26	196	<0.001
				(57.4%)	(34.2%)	(12.0%)	
Insulin	0	0	0	0	76	76	<0.001
					(100%)	(4.6%)	
Frusemide	54	43	14	39	23	173	<0.001
	(8.2%)	(9.3%)	(9.3%)	(13.2%)	(30.3%)	(10.6%)	

Serum creatinine was unavailable on 7*, 3†, 3‡, 1§ and 1^||^ patients therefore eGFR could not be calculated. CHD = coronary heart disease; ACE = angiotensin converting enzyme; ARB = angiotensin receptor blocker; eGFR = estimated glomerular filtration rate. Continuous variables are presented as median and inter-quartile range and assessed using the Kruskal Wallis test. Nominal variables are presented as number and percent and analysed using the Chi-squared test.

Key to patient groups:

1: No diabetes; 2: Impaired fasting glucose; 3: Non-medicated diabetes; 4: Diabetes prescribed oral hypoglycaemics only; 5: Diabetes prescribed insulin.

### Relationship between risk factors, presentation, impaired fasting glucose, diabetes and its management

At entry 372 (22.7%) patients were receiving treatment for type 2 diabetes by oral hypoglycaemics alone (n = 296; 18.1%) or insulin (n = 76; 4.6%). Fasting blood glucose concentrations (≥7 mM) suggested diabetes in a further 150 (9.2%) patients who were on no medications for diabetes. A further 460 (28.1%) patients had fasting blood glucose concentrations between 5.6 and 6.9 mM which we defined as impaired fasting glucose. The other 655 (40.0%) patients had a normal fasting blood glucose concentration (<5.6 mM). Diabetes category was significantly associated with age, hypertension, CHD, eGFR, and prescription of statins, aspirin, beta blockers, CCBs, ACE inhibitors, ARBs and frusemide (Table 1). Patients with diabetes prescribed insulin were younger, had lower eGFR and more commonly had a history of hypertension and CHD than those with no diabetes (Table 1). Patients with diabetes prescribed insulin were also more likely to be prescribed statins, beta blockers, CCBs, ACE inhibitors and frusemide than those without diabetes (Table 1).

No difference in the prevalence of asymptomatic or symptomatic carotid stenosis was observed between the diabetes categories. Patients with non-medicated diabetes were more likely to present with aortic or peripheral artery aneurysms. Patients with diabetes who were prescribed insulin more frequently presented with mild limb peripheral artery disease and critical limb ischaemia.

### Relationship between impaired fasting glucose, diabetes and its management with mortality

**Figure 1:**
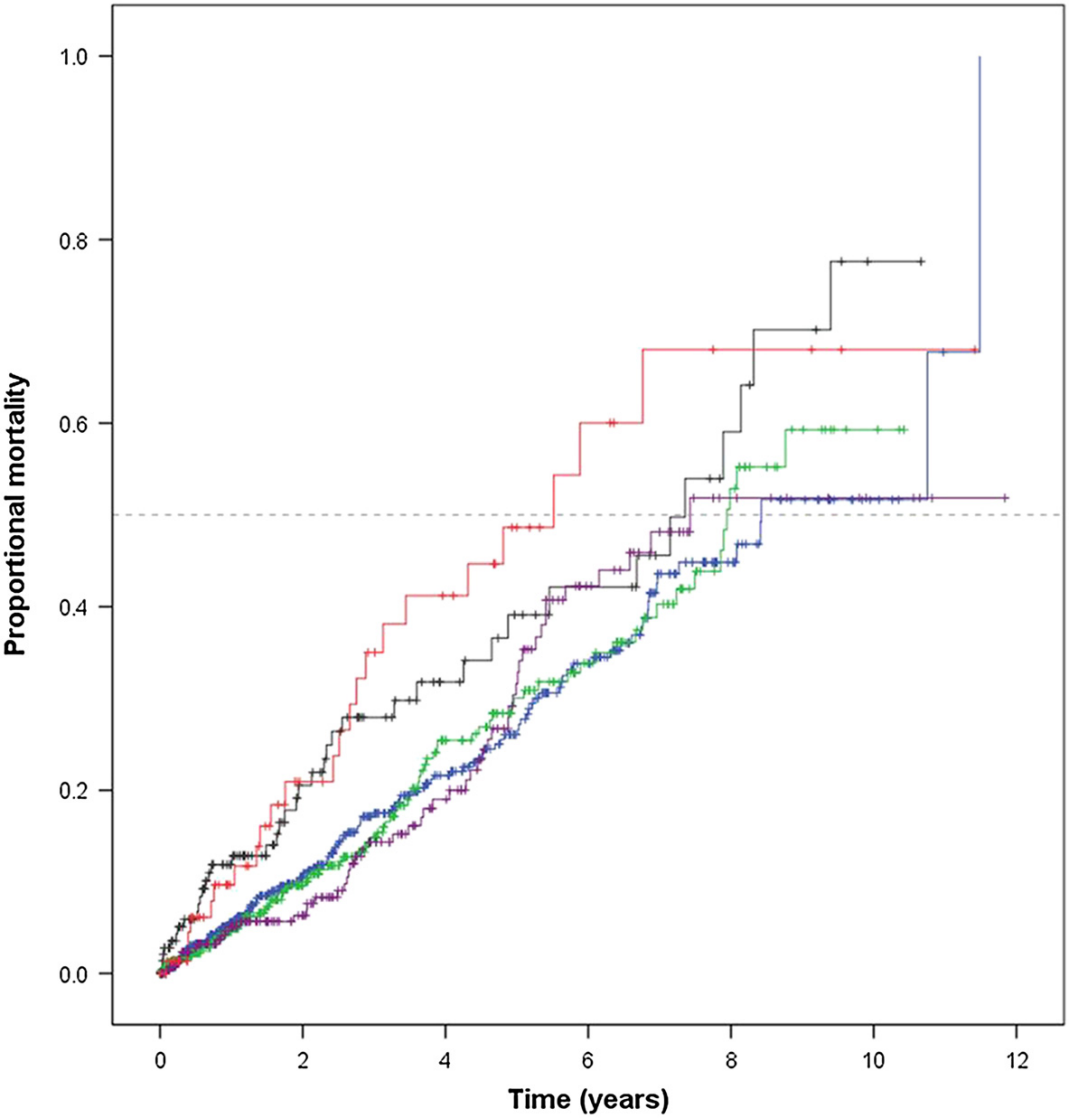
**Kaplan Meier curves showing the cumulative proportional mortality in relation to diabetes categories.** Lines represent mortality for subjects grouped by diabetes categories. The blue, green, black, purple and red lines represent no diabetes, impaired fasting glucose, non-medicated diabetes, diabetes prescribed oral hypoglycaemics only and diabetes prescribed insulin, respectively. Vertical lines represent subjects censored at loss to follow-up.

Median follow-up time was 1.7 (interquartile range 0.5-4.1) years. Three hundred and 17 (19.4%) patients died during the follow up period. Kaplan Meier analyses suggested that mortality was greatest in patients with diabetes prescribed insulin who had an estimated mortality of 20.9% at 2 years (Figure 1). Estimated mortality at 2 years in these patients was greater than that of patients with no diabetes (10.6%; p = 0.004), impaired fasting glucose (9.6%; p = 0.006) and diabetes prescribed oral hypoglycaemics only (6.3%; p = 0.008). Estimated mortality at 2 years in patients with non-medicated diabetes was similar to that of patients with diabetes prescribed insulin (20.5%; p = 0.611). Cox proportional hazard analyses suggested that, depending on the other risk factors adjusted for, mortality increased 1.6-1.7 fold in patients with non-medicated diabetes compared to patients with no diabetes (Table 2). Similarly patients with diabetes prescribed insulin had an increased mortality of 2.0-2.9 fold (Table 2). Patients with impaired fasting glucose and diabetes prescribed oral hypoglycaemics only had similar mortality to that of patients with no diabetes (Table 2).

**Table 2:** **Association of impaired fasting glucose, diabetes and their treatment with mortality in 1637 patients with peripheral artery disease across 4 regression models**

	**Model 1**		**Model 2**		**Model 3**		**Model 4**	
**Patient group**	**HR**	**P value**	**HR**	**P value**	**HR**	**P value**	**HR**	**P value**
	**(95% CI)**		**(95% CI)**		**(95% CI)**		**(95% CI)**	
No diabetes	1.00		1.00		1.00		1.00	
	(reference)		(reference)		(reference)		(reference)	
Impaired fasting glucose	1.02	0.901	0.99	0.967	1.01	0.949	1.01	0.925
	(0.77-1.35)		(0.75-1.32)		(0.76-1.35)		(0.76-1.35)	
Non-medicated diabetes	1.73	0.003	1.63	0.009	1.64	0.008	1.62	0.011
	(1.21-2.48)		(1.13-2.35)		(1.14-2.37)		(1.12-2.34)	
Diabetes prescribed oral hypoglycaemics only	1.11	0.522	1.03	0.870	1.01	0.935	1.03	0.874
	(0.80-1.54)		(0.74-1.43)		(0.72-1.43)		(0.73-1.45)	
Diabetes prescribed insulin	2.94	<0.001	2.28	0.001	1.98	0.006	1.97	0.007
	(1.86-4.66)		(1.42-3.66)		(1.21-3.23)		(1.20-3.23)	

Models include the following covariates.

Model 1: Age and sex.

Model 2: Age, sex, hypertension, ever smoking, coronary heart disease and presenting complaint.

Model 3: Age, sex, hypertension, ever smoking, coronary heart disease, presenting complaint, statin prescription, aspirin prescription, other anti-platelet prescription, beta blocker prescription, calcium channel blocker prescription, angiotensin converting enzyme inhibitor prescription, angiotensin receptor blocker prescription and frusemide prescription.

Model 4: Age, sex, hypertension, ever smoking, coronary heart disease, presenting complaint, statin prescription, aspirin prescription, other anti-platelet prescription, beta blocker prescription, calcium channel blocker prescription, angiotensin converting enzyme inhibitor prescription, angiotensin receptor blocker prescription, frusemide prescription and eGFR. Please note, 15 patients with missing serum creatinine values were excluded from this analysis.

### Relationship between impaired fasting glucose, diabetes and its management with peripheral artery interventions

**Figure 2:**
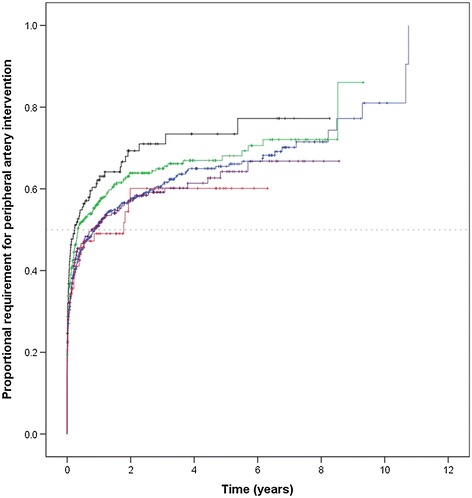
**Kaplan Meier curves showing the cumulative proportional requirement for peripheral artery intervention in relation to diabetes categories.** Lines represent requirement for peripheral artery intervention for subjects grouped by diabetes categories. The blue, green, black, purple and red lines represent no diabetes, impaired fasting glucose, non-medicated diabetes, diabetes prescribed oral hypoglycaemics only and diabetes prescribed insulin, respectively. Vertical lines represent subjects censored at loss to follow-up.

During follow up, 963 (58.8%) patients required a peripheral artery intervention. Patients with non-medicated diabetes were more likely to require a peripheral artery intervention during follow-up (Figure 2). Kaplan Meier analyses suggested that by two years after entry 69.3% of these patients had required a peripheral artery intervention. This intervention requirement was significantly greater than for patients with no diabetes (57.4%; p = 0.008) and those with diabetes prescribed oral hypoglycaemics only (57.1%; p = 0.018), and almost significantly higher than for patients prescribed insulin (60.1%; p = 0.063). The intervention rate in patients with impaired fasting glucose (63.9%; p = 0.210) was similar to that of patients with non-medicated diabetes (Figure 2). Cox proportional hazard analyses suggested that the requirement for peripheral artery intervention was approximately 1.3-fold greater in patients with non-medicated diabetes compared to those with no diabetes in different models which included adjustment for other risk factors (Table 3). The requirement for peripheral artery intervention was similar in patients with impaired fasting glucose, diabetes prescribed oral hypoglycaemics only, diabetes prescribed insulin and no diabetes (Table 3).

**Table 3:** **Association of impaired fasting glucose, diabetes and their treatment with requirement for peripheral artery intervention in 1637 peripheral artery disease patients across 4 regression models**

	**Model 1**		**Model 2**		**Model 3**		**Model 4**	
**Patient group**	**HR**	**P value**	**HR**	**P value**	**HR**	**P value**	**HR**	**P value**
	**(95% CI)**		**(95% CI)**		**(95% CI)**		**(95% CI)**	
No diabetes	1.00		1.00		1.00		1.00	
	(reference)		(reference)		(reference)		(reference)	
Impaired fasting glucose	1.14	0.099	1.13	0.138	1.11	0.195	1.13	0.141
	(0.98-1.33)		(0.96-1.32)		(0.95-1.30)		(0.96-1.32)	
Non-medicated diabetes	1.33	0.011	1.31	0.016	1.29	0.026	1.31	0.020
	(1.07-1.66)		(1.05-1.64)		(1.03-1.61)		(1.04-1.64)	
Diabetes prescribed oral hypoglycaemics only	0.98	0.790	0.96	0.682	0.93	0.434	0.94	0.496
	(0.81-1.17)		(0.80-1.16)		(0.77-1.12)		(0.77-1.13)	
Diabetes prescribed insulin	0.90	0.542	0.79	0.179	0.82	0.260	0.83	0.299
	(0.65-1.25)		(0.57-1.11)		(0.58-1.16)		(0.59-1.18)	

Models include the following covariates.

Model 1: Age and sex.

Model 2: Age, sex, hypertension, ever smoking, coronary heart disease and presenting complaint.

Model 3: Age, sex, hypertension, ever smoking, coronary heart disease, presenting complaint, statin prescription, aspirin prescription, other anti-platelet prescription, beta blocker prescription, calcium channel blocker prescription, angiotensin converting enzyme inhibitor prescription, angiotensin receptor blocker prescription and frusemide prescription.

Model 4: Age, sex, hypertension, ever smoking, coronary heart disease, presenting complaint, statin prescription, aspirin prescription, other anti-platelet prescription, beta blocker prescription, calcium channel blocker prescription, angiotensin converting enzyme inhibitor prescription, angiotensin receptor blocker prescription, frusemide prescription and eGFR. 15 patients with missing serum creatinine values were excluded from this analysis.

## Discussion

The current study has a number of important findings. Firstly, impaired fasting glucose and diabetes are common in patients with PAD. Only 40% of patients had normal fasting glucose concentrations at entry and were not currently receiving medication for diabetes. Approximately 28% of patients had fasting glucose concentrations within the current definition for impaired fasting glucose [19,20]. Approximately 23% of patients had been diagnosed with diabetes and were prescribed oral hypoglycaemics and/ or insulin. A further 9% of patients had fasting glucose concentrations in the diabetes range and were currently not prescribed oral hypoglycaemics or insulin. Secondly, patients with non-medicated diabetes at entry had higher mortality and increased requirement for peripheral artery intervention compared to patients without diabetes. Thirdly, patients with diabetes prescribed hypoglycaemics only had a similar mortality to patients with no diabetes. Conversely patients with diabetes receiving insulin had a ~2-3 fold increased risk of mortality compared to patients with no diabetes.

PAD patients with non-medicated diabetes showed higher rates of mortality and peripheral artery intervention compared to patients with normal fasting glucose. The exact reasons for this remain unclear but, may reflect poor glycaemic control compared to patients receiving oral hypoglycaemics. Previous data suggest that poor diabetes control, evidenced by high HbA1c concentrations, is positively associated with major adverse events in patients with established cardiovascular disease [21-23]. The reasons that these patients were not receiving medications for diabetes were not investigated here, although dietary glycaemic management, a normal or mildly impaired previous oral glucose tolerance test or newly identified diabetes may be likely explanations. We also noted that these patients were less likely to be receiving some other medications like statins, aspirin and ACE inhibitors than patients with diabetes on medication indicating that these patients were receiving less secondary cardiovascular protection. It is possible these patients may have been less willing to visit their local medical practitioner, or comply with treatment advice. Two of the hospitals from which participants were recruited have large rural Australian catchment areas. A recent audit of rural aged care facilities demonstrated that one third of residents with diabetes were managed by diet alone and only 41% were managed according to current diabetes guidelines [24]. Similar findings have been reported in other rural health care audits [25]. It is therefore possible that the outcomes of the current study are reflective of management strategies used by rural physicians although we did not directly study this.

Patients prescribed oral hypoglycaemics in the current study had outcomes similar to those of patients that did not have diabetes. The mortality of these patients was similar to that of patients without diabetes. Previous data indicate that oral hypoglycaemics, particularly metformin, reduce the incidence of major adverse events in patients with cardiovascular disease [26,27]. More recent evidence suggests that metformin might also improve the outcome of cancer which is a common co-morbidity in cardiovascular disease patients [28]. In contrast patients prescribed insulin had a 2-3fold increased mortality in the current study. These findings are in keeping with other studies which have highlighted the high cardiovascular event rate of diabetes patients on insulin [29-32]. It has even been suggested that insulin use may increase event rate and recent trials attempting to achieve tighter glucose control have reported worse rather than improved outcomes [33,34]. The poor outcome of patients requiring insulin reported in the current study is likely multifactorial, and in part probably reflects their poor glycaemic control. Recent data demonstrate marked differences in the expression of several genes governing atherogenesis and plaque instability in arterial biopsies collected from PAD patients with good and poor glycaemic control [35]. The complications of PAD patients with insulin-controlled diabetes appear to be greater although the exact reasons for this requires further study.

The fasting blood glucose range which should be used to define pre-diabetes is controversial [20,36,37]. Current guidelines suggest that fasting blood glucose in the range of 5.6 to 6.9 mM should be defined as impaired fasting glucose and this was employed in the current study [20,36]. The frequency of impaired fasting glucose reported in the current study (28%) is greater than that reported (17%) in a recent study which focussed on patients with lower limb athero-thrombosis [13]. In the latter study patients also underwent oral glucose tolerance tests and it was noted that impaired fasting glucose did not define the same group of subjects as does an impaired response to a glucose tolerance test [14]. Glucose tolerance tests were not performed in the current study. It has been suggested that the diagnosis of impaired fasting glucose overestimates the burden of dysglycaemia [20,36]. It is relevant to note that PAD patients with impaired fasting glucose had similar mortality and requirement for peripheral artery intervention as patients with normal fasting glucose in this study.

### Study strengths and limitations

The current study has a number of strengths and weakness. Strengths include the large sample size, the adjustment for many potential confounding factors and the large range of different peripheral artery disease complaints studied. Weaknesses include the absence of information on oral glucose tolerance tests, dietary management of diabetes or HbA1C. The overall follow-up of the patients was also relatively short at a median of approximately 2 years. Importantly, we were only able to collect clinical characteristics of the included patients at the time of recruitment. Hall and colleagues recently reported that altering anti-glycaemic medication regime during a 3.5 year follow-up significantly influences cardiovascular outcomes in patients with diabetes [31]. However, the design of the current study limited the ability to accommodate changes in risk factors which may have influenced cardiovascular outcome in our patient cohorts. Finally, the cause of death was not available and it is therefore possible that other factors may have contributed to overall mortality in the current study, complicating interpretation of our findings. Future studies are required to specifically investigate the relationship between diabetes presentation, management and cardiovascular mortality in the northern Australian PAD population.

### Study conclusions

The findings of this work have important implications. Firstly dysglycaemia is very common in PAD patients. Screening newly referred patients for diabetes and where appropriate referring them to a diabetes specialist would appear appropriate as previously suggested [38]. Secondly, patients with fasting blood glucose ≥7 mM not currently receiving medication for diabetes are at increased risk of major adverse events and may benefit from more intensive management. Patients with established diabetes receiving insulin are at particularly high risk of major adverse events which should be considered in decisions about surgical interventions.

## Additional file

Additional file 1:
**Supplement 1.** Expanded Materials and Methods.

## Abbreviations

PAD: Peripheral artery disease
CHD: Coronary heart disease
