# Supplementary material for: Association of impaired fasting glucose, diabetes and their management with the presentation and outcome of peripheral artery disease: a cohort study
Source: Cardiovasc Diabetol. 2014 Nov 1;13:147. doi: 10.1186/s12933-014-0147-2 (PMC4230372; doi:10.1186/s12933-014-0147-2)
Supplement: Additional file 1: — Supplement 1. Expanded Materials and Methods. [file 12933_2014_147_MOESM1_ESM.pdf]

SUPPLEMENT 1: EXPANDED MATERIALS AND METHODS

Study design and sample size: This study was part of on-going prospective cohort investigation of patients with peripheral vascular disease aimed at assessing risk predictors of peripheral vascular disease presence and outcome commencing in 2002, as previously described [1,2]. No formal sample size calculation was performed. Monte-Carlo simulations suggest that a multivariate regression model is powered sufficiently when 10 outcome events per degree of freedom of the predictor variables are observed [3]. We estimated that mortality at one year would be approximately 10% and planned to adjust for up to 15 variables (Age, sex, hypertension, ever smoking, coronary heart disease (CHD), presenting complaint, statin prescription, aspirin prescription, other anti-platelet prescription, beta blocker prescription, calcium channel blocker (CCB) prescription, angiotensin converting enzyme (ACE) inhibitor prescription, angiotensin receptor blocker (ARB) prescription, frusemide prescription and estimated glomerular filtration rate (eGFR)) in our regression model. Based on these estimates we felt that a sample size of approximately 1600 patients would be well powered to examine the association of diabetes with mortality.

Patients: Patients were recruited from in and out-patient vascular services at The Townsville Hospital, The Mater Hospital Townsville and The Royal Brisbane and Women's Hospital, Australia. Patients with all types of peripheral vascular disease were considered for inclusion as previously described [1,2]. Inclusion criteria for the current study included a diagnosis of PAD, the assessment of fasting blood glucose and at least one follow-up assessment as an in or out-patient. This study was conducted in accordance with the Declaration of Helsinki. Ethical approval for the study was granted by the local Institutional Ethics Committees at The Townsville Hospital, The Mater Hospital Townsville, The Royal Brisbane and Women's

Hospital and James Cook University (61/05, MHS2006-01, H2196, 2007/004, 12/QTHS/202, MHS20140114-01, H5206, 13/ QTHS/125). Written informed consent was obtained from participants.

Definition of PAD complaints

PAD complaints were defined using the following criteria [1,2]: a) Asymptomatic carotid artery stenosis: Defined as the presence of ≥50% stenosis or occlusion of at least one carotid artery identified by carotid duplex but the absence of physician confirmed symptoms of focal transient ischemic attack, amaurosis fugax or stroke [4]; b) Mild lower limb or upper limb peripheral athero-thrombosis: This included patients with intermittent claudication, atypical or no symptoms with clinical evidence of lower or upper limb ischaemia but not critical lower limb ischaemia. Limb peripheral athero-thrombosis was confirmed by a vascular specialist by identification of absence of lower or upper limb pulses, ankle brachial pressure index <0.9 and/or significant stenosis (>50%) or occlusion of lower or upper limb arteries on computed tomographic angiography or duplex imaging [5,6]. c) Aneurysm of the aorta or peripheral arteries: Abdominal aortic aneurysm was defined as maximum infra-renal aortic diameter ≥30mm [5-7]. Iliac artery aneurysm was defined by common or internal iliac artery diameters ≥15 and ≥8mm, respectively. Femoral artery aneurysm was defined by common femoral or superficial femoral artery diameter of ≥15mm. Popliteal artery aneurysm was defined as popliteal artery diameter ≥9mm [8]; d) Symptomatic carotid artery stenosis: Defined as the presence of ≥50% stenosis or occlusion of at least one carotid artery identified with carotid duplex with the presence of physician confirmed symptoms of focal transient ischemic attack, amaurosis fugax or stroke [4]; e) Critical lower limb ischaemia: Rest pain, arterial ulcer or gangrene of the leg due to athero-thrombosis of the lower limb. Peripheral athero-thrombosis was confirmed as detailed above [5,6]. For patients with more than one

presenting complaint classification was determined by the complaint which was deemed most severe.

Assessment of fasting blood glucose and diabetes

Patients were asked if they were receiving medications for the treatment of diabetes, specifically oral hypoglycaemics, such as metformin, sulphonylureas, thiazolidinediones and alpha-glucosidase inhibitors, or insulin. Patients provided blood samples after an overnight fast for assessment of blood glucose as part of their clinical care. Blood was collected into SST tubes and serum glucose analysed using an automated assay using an Integra 800 chemistry analyzer (Roche Diagnostics, Basel, Switzerland) in a pathology laboratory. The assay relies on the rate of NADPH formation which is measured photometrically. The assay undergoes regular quality control assessments required for its use in clinical care. A commercial quality control sample is run on a daily basis and results entered and accepted into a laboratory quality program before patient samples can be analysed. In patients who were not currently receiving oral hypoglycaemic medication or insulin fasting blood glucose of 5.6-6.9 and ≥ 7.0mM were used to define impaired fasting glucose and diabetes, respectively [9,10]. Utilising the information collected on medical treatment of diabetes and blood glucose patients were allocated into the following groups for the current analysis:

Follow-up: Patients were followed up through attendance at out-patient clinics and /or as an in-patient as part of their normal medical care as previously described [1,2]. Patients with limb athero-thrombosis and carotid artery stenosis were generally reviewed 6 months after their initial assessment and then yearly unless symptoms or imaging findings changed [3-5]. Patients with small aneurysms or large aneurysms that had been repaired were followed up

yearly or 6 monthly if the aneurysm was nearing a diameter at which intervention was indicated [6,7].

Recording of outcome data: The primary outcome was mortality. The secondary outcome was requirement for a peripheral artery intervention (including peripheral angioplasty, peripheral stent placement, open peripheral revascularization, open carotid revascularisation, aortic aneurysm repair or other peripheral aneurysm repair). Outcome data was recorded during clinical reviews on prospectively defined report forms and entered into an Access database (Microsoft Incorporated, Redmond, Washington, USA). Charts and hospital electronic records of all patients were reviewed at least once for the identification and date of other outcome events by a vascular specialist or clinical researcher. Where uncertainty about outcome data was present a discussion occurred between the clinical researcher and vascular specialist and a consensus was reached. For peripheral artery intervention assessments, patients were censored at the time of the first intervention or at the date of last in/out patient review or death if no intervention was required.

Statistical analyses: Quantitative data were not normally distributed and therefore are presented as median and inter-quartile range and assessed by non-parametric tests. Nominal data are presented as number and percentages. The association of diabetes categories with the clinical presentation and risk factors of the patients was assessed using Kruskal Wallis and chi-squared tests. The associations of diabetes categories with death and requirement for peripheral artery intervention were assessed using Kaplan Meier estimates, log rank test and Cox proportional hazard analyses. Cox proportional hazard analyses were adjusted for age and sex; age, sex, hypertension, ever smoking, CHD and presenting complaint; age, sex, hypertension, ever smoking, CHD, presenting complaint, statin prescription, aspirin

prescription, other anti-platelet prescription, beta blocker prescription, CCB prescription, ACE inhibitor prescription, ARB prescription and frusemide prescription; age, sex, hypertension, ever smoking, CHD, presenting complaint, statin prescription, aspirin prescription, other anti-platelet prescription, beta blocker prescription, CCB prescription, ACE inhibitor prescription, ARB prescription, frusemide prescription and eGFR in four different models. These covariates were included as they are recognised determinants of outcome for patients with cardiovascular disease

REFERENCES

1. Golledge J, Cronin O, Iyer V et al. Body mass index is inversely associated with mortality in patients with peripheral vascular disease. Atherosclerosis. 2013;229:549-55.

2. Golledge J, Ewels C, Muller R et al. Association of chronic kidney disease categories defined with different formulae with major adverse events in patients with peripheral vascular disease. Atherosclerosis. 2014;232:289-97.

3. Peduzzi P, Concato J, Kemper E et al. A simulation study of the number of events per variable in logistic regression analysis. J Clin Epidemiol. 1996;49:1373-1379.

4. Palamuthusingam D, Quigley F, Golledge J. Implications of the finding of no significant carotid stenosis based on data from a regional Australian vascular unit. Ann Vasc Surg. 2011;25:1050-1056.

5. Parr A, Buttner P, Shahzad A et al. Relation of infra-renal abdominal aortic calcific deposits and cardiovascular events in patients with peripheral artery disease. Am J Cardiol. 2010;105:895-899.

6. Golledge J, Jayalath R, Oliver L et al. Relationship between CT anthropometric measurements, adipokines and abdominal aortic calcification. Atherosclerosis. 2008;197:428-434.

7. Parr A, McCann M, Bradshaw B et al. Thrombus volume is associated with cardiovascular events and aneurysm growth in patients who have abdominal aortic aneurysms. J Vasc Surg. 2011;53:28-35.

8. Magee R, Quigley F, McCann M et al. Growth and risk factors for expansion of dilated popliteal arteries. Eur J Vasc Endovasc Surg. 2010;39:606-611.

9. Genuth S, Alberti KG, Bennett P et al, the Expert Committee on the Diagnosis and Classification of Diabetes Mellitus: Follow-up report on the diagnosis of diabetes mellitus. Diabetes Care 2003;26:3160–3167.

10. Shaw JE, Zimmet PZ, Alberti KG. Point: Impaired fasting glucose: The case for the new American diabetes association criterion. Diabetes Care 2006;29:1170-2
